# The MYEOV-MYC association promotes oncogenic miR-17/93-5p expression in pancreatic ductal adenocarcinoma

**DOI:** 10.1038/s41419-021-04387-z

**Published:** 2021-12-20

**Authors:** Hongzhang Shen, Fuqiang Ye, Dongchao Xu, Liangliang Fang, Xiaofeng Zhang, Juanjuan Zhu

**Affiliations:** 1 Department of Gastroenterology, Affiliated Hangzhou First People’s Hospital, Zhejiang University School of Medicine, Hangzhou, China; 2 Huadong Research Institute for Medicine and Biotechniques, Nanjing, China; 3 The First School of Clinical Medicine, Nanjing Medical University, Nanjing, China; 4 School of Life Science and Technology, China Pharmaceutical University, Nanjing, China

**Keywords:** Cell biology, Pancreatic cancer

## Abstract

Pancreatic ductal adenocarcinoma (PDAC) is a highly lethal malignancy worldwide. As metastasis and malignant progression are primarily responsible for the poor clinical outcomes of PDAC, identifying key genes involved in these processes and the underlying molecular mechanisms of PDAC is vital. In this study, by analyzing TCGA PDAC data and matched GTEx data, we found that MYEOV expression is associated with poor survival in PDAC patients and higher in carcinoma tissues than in healthy tissues. Elevated levels of MYEOV led to enhanced cell proliferation, invasion and migration in vitro and in vivo. Transcriptome analysis results revealed that MYEOV mediates global alterations in gene expression profiles in PDAC cells. MiRNA-seq analysis showed that MYEOV regulates the expression levels of miR-17-5p and miR-93-5p, and its depletion resulted in reduced cell proliferation, invasion and migration, as observed in MYEOV-knockdown PDAC cells. These effects are likely due to the ability of MYEOV to regulate enrichment of the transcription factor MYC at the gene promoter regions of the two miRNAs. Furthermore, we identified a complex containing MYEOV and MYC in the nucleus, providing additional evidence for the association of MYEOV with MYC. Taken together, our results suggest that MYEOV promotes oncogenic miR-17/93-5p expression by associating with MYC, contributing to PDAC progression.

## Introduction

Pancreatic ductal adenocarcinoma (PDAC) is a highly lethal malignancy originating in the human pancreas, with fewer than 7% of patients surviving past 5 years [1]. PDAC is a major clinical problem because it has the worst prognosis among all cancers [2]. This poor prognosis is largely due to a delayed diagnosis owing to the lack of any symptoms in its early stages of development, with a PDAC diagnosis often considered to be a “death sentence” to patients [3, 4]. Due to the highly advanced stage and the extent of metastasis to distant sites at the time of diagnosis, PDAC is clinically challenging to treat [5]. Despite substantial advancements in understanding the molecular progression of PDAC, the pathogenesis and mechanisms of PDAC remain unclear, highlighting the need to identify novel targets that may contribute to the highly malignant phenotype of this cancer.

Myeloma overexpressed gene (MYEOV) is a candidate proto-oncogene [6] that is closely associated with gene recombination in many malignant tumors [7–9]. MYEOV is predominantly overexpressed and promotes tumorigenesis in various human cancers, including breast cancer, gastric cancer, colon cancer and non-small cell lung cancer (NSCLC) [10–14]. Similarly, for the first time, we demonstrated herein that MYEOV expression is significantly upregulated in PDAC. Although the dysregulated expression of MYEOV in cancer patients has been associated with its tumorigenic properties, the molecular mechanisms underlying MYEOV-mediated tumorigenesis, particularly in PDAC, remain largely unknown.

MicroRNAs (miRNAs) are involved in PDAC pathobiology [15]. Accumulating data have shown that abnormally expressed miRNAs in PDAC are closely associated with tumor occurrence. Many of these miRNAs have been identified as tumor suppressors or onco-miRNAs that modulate PDAC cell initiation, promotion, metastasis and chemoresistance [16, 17]. Castellano et al. showed that miR-100 and miR-125b synergize with TGF-β to control PDAC tumorigenesis [18]. In another study, the tumor suppressor gene miR-489 inhibited migration and metastasis by targeting the extracellular matrix factors ADAM9 and MMP7 in PDAC [19]. However, the potential interaction between MYEOV and PDAC-related miRNAs requires further investigation.

The oncogene c-MYC (hereafter MYC) governs many crucial cellular functions, including proliferation, apoptosis and metabolism [20, 21]. Dysregulation of MYC expression is associated with the pathogenesis of many human cancers [22, 23]. As a transcription factor, MYC triggers the expression of specific genes, including a broad repertoire of miRNAs, to promote cell growth and proliferation [22, 24]. In B-cell lymphomas, MYC can directly regulate expression of the miR-17-92 gene cluster and inhibit apoptosis [25]. MYC can also regulate tumor progression by inhibiting the expression of other miRNAs, such as the let-7 miRNA family, miR-15a/16-1, miR-26a and miR-34a [26, 27]. Therefore, miRNA expression profiles induced by MYC could play an important role in tumor progression.

MYC is predicted to be the most affected transcriptional factor in response to MYEOV knockdown [28]. Therefore, we speculated that, by regulating MYC activity, an MYEOV-mediated regulatory effect on the expression of specific miRNAs might occur, thus mediating PDAC oncogenesis. The present study revealed that MYEOV expression is significantly upregulated in PDAC cells and patients and associated with poor patient survival. MYEOV depletion restricted the growth of PDAC cells. Importantly, we showed that MYC-induced upregulation of miR-17-5p and miR-93-5p is involved in MYEOV-mediated PDAC oncogenesis. Moreover, MYC enrichment at miR-17/93 gene promoter regions was regulated by MYEOV via the MYEOV-MYC association. In summary, we discovered a novel regulatory role of MYEOV in PDAC progression

## Materials and methods

### Tissues and cell lines

Nineteen pairs of formalin-fixed paraffin-embedded (FFPE) pancreatic carcinoma tissues and adjacent normal tissues were obtained from the Affiliated Hangzhou First People’s Hospital, Zhejiang University School of Medicine (Hangzhou, China). Informed consent was obtained from each participant, and the sampling protocols were performed in accordance with the operational guidelines. The clinical characteristics of the patients included in this study are listed in Table S1. This study was approved by the Ethics Committee of Hangzhou First People’s Hospital. The human PDAC cell lines MIAPaCa-2, BxPC-3, PANC-1 and AsPC-1, as well as normal human pancreatic duct epithelial (HPDE) cells, were obtained from ATCC (Manassas, VA, USA) and cultured according to the manufacturer’s instructions.

### Plasmid construction and siRNA construction, transfection and infection

To overexpress MYEOV, we cloned full-length *Homo sapiens* MYEOV sequences (NM_001293291.2) into the vector pcDNA3.0. To inhibit MYEOV, an siRNA against human MYEOV (Stealth RNAi: siMYEOV-1, HSS120166; siMYEOV-2, HSS120167) and negative control siRNA (Stealth RNAi: siNC, #12935-300) were purchased from Life Technologies (Invitrogen, Carlsbad, CA). To overexpress and inhibit miR-17-5p and miR-93-5p, miRNA mimics and inhibitors were purchased from Ribobio (Guangzhou, China). Plasmids, siRNAs, mimics and inhibitors were transfected into PDAC cells using Lipofectamine 3000 (Invitrogen) according to the manufacturer’s protocol.

To obtain cell lines stably overexpressing MYEOV, MYEOV was cloned into the lentivirus plasmid Pez-Lv105. For stable knockdown cells, the shRNA sequence targeting MYEOV was ligated into the psi-LVRU6MP lentivirus plasmid. Recombinant lentivirus infection and screening were performed according to the manufacturer’s protocol (GeneCopoeia^TM^, Rockville, MD).

### RNA isolation and quantitative reverse transcription PCR

Total RNA was extracted from cells using TRIzol reagent as previously described [29]. For mRNA detection, cDNA was synthesized from 1 μg of total RNA using an Oligo (dT) reverse transcription primer and a HiScript® III 1st Strand cDNA Synthesis kit (Vazyme, Nanjing, China). For miRNA detection, cDNA was generated using 1 μg of total RNA and a specific Bulge-Loop^TM^ miRNA RT primer (Ribobio). Quantitative polymerase chain reaction (qPCR) was performed using ChamQ SYBR qPCR Master Mix (Vazyme). Quantitation of the relative expression levels of the target genes was performed in triplicate and calculated using the 2^−ΔΔCT^ method. GAPDH and U6 [29–31] were used as endogenous controls to normalize mRNA and miRNA expression, respectively.

### Immunoprecipitation and Western blot assays

For the immunoprecipitation (IP) assay, cells were lysed in lysis buffer (50 mM Tris-Cl [pH 7.4], 150 mM NaCl, 0.5% Triton X-100, and 1 mM EDTA) supplemented with protease inhibitor cocktail (Roche, Switzerland). The lysates were incubated with the appropriate antibodies for 4 h overnight at 4 °C before protein A/G agarose beads were added and incubated with the samples for 2 h. The beads were then washed four times with lysis buffer and eluted for 5 min with SDS loading buffer. Subsequently, the immunoprecipitated proteins were then subjected to Western blot analysis as described previously [32]. Anti-c-MYC (#5605; Cell Signaling Technology, USA), anti-β-actin (A5441; Sigma-Aldrich, USA) and anti-MYEOV (ab121387; Abcam, England) antibodies were used for Western blotting.

### Immunohistochemistry

All the samples were fixed in 4% paraformaldehyde, embedded in paraffin, sliced into sections, and placed on adhesion microscope slides. The sections were then subjected to immunohistochemical (IHC) staining according to standard procedures, with anti-MYEOV (HPA012949; Sigma) used as the primary antibody.

### Immunofluorescence and confocal microscopy

Confocal microscopy was performed as previously described [33]. Briefly, cells seeded onto glass coverslips were fixed with 4% paraformaldehyde in PBS for 20 min, permeabilized with 0.25% Triton X-100 and blocked with 5% bovine serum albumin at room temperature. The cells were subsequently incubated with the indicated primary antibodies, followed by staining with AlexaFluor488- and Cyanin3-conjugated secondary antibodies (both from ThermoFisher, USA) for immunofluorescence detection. Nuclei were counterstained with DAPI (Sigma-Aldrich), and the slides were mounted with fluorescence mounting medium (Dako, Denmark). Images of the cells were captured using a Carl Zeiss LSM700 laser scanning confocal microscope under a 64× oil objective.

### Colony formation and cell proliferation assays

For colony formation assays, transfected cells were plated in six-well plates in triplicate at a density of 500 cells per well. The cells were allowed to grow for two weeks at 37 °C until colonies were visible. To visualize the colonies, they were fixed in 4% paraformaldehyde for 15 min and then stained with a 0.1% crystal violet solution (Sigma-Aldrich). The cell proliferation rate was determined using the Cell Counting Kit-8 (CCK-8) assay (MedChemExpress, China) according to the manufacturer’s protocol. After 48 h of transfection, AsPC-1 and PANC-1 cells (2 × 10^3^) were seeded in 96-well plates (100 μL). Subsequently, 10 μL of CCK-8 solution was added to each well, and the plates were incubated at 37 °C for an additional 2 h. An automatic microplate reader (Tecan, Austria) was used to measure the absorbance of each well at a wavelength of 450 nm. Additionally, a standard curve was established to calculate the cell numbers. Each experiment was repeated at least three times.

### Transwell invasion assay

The treated cells were cultured in serum-free medium for 24 h. Next, 5 × 10^4^ serum-starved cells were added to the top of a 24-well Millipore Transwell chamber (Millipore, USA) coated with diluted Matrigel (BD Biosciences, Germany), and 600 μL of culture medium containing 20% FBS was added to the lower chamber. After 24 h, the cells located in the lower chamber were stained with a 0.1% crystal violet solution for microscopic analysis.

### Wound scratch assay

A scratch was made with a sterile P200 pipette tip in the center of each well of a six-well plate cultured with transfected cells. The medium was replaced with serum-free medium, and the transfected cells were incubated for 24 h. The ability of the cells to migrate across the wound was determined by comparing the 0- and 24-h phase contrast micrographs of three marked positions. The wound area was measured, and the percentage of wound healing was estimated using ImageJ (v 1.8.0).

### Flow cytometry assay

For apoptosis analysis, AsPC-1 and PANC-1 cells transfected with siRNA after serum starvation for 24 h were harvested by trypsinization. After they were double stained with FITC-Annexin V and propidium iodide (PI; BD Biosciences), the cells were subjected to flow cytometry using the Attune NxT flow cytometer (Life Technologies) and analyzed using FlowJo software (v10.0.7). The cells were divided into four categories: viable, dead, early apoptotic and apoptotic cells. All assays were independently performed at least three times.

### Tumor xenograft assay

The animal experiments and care were approved by the Animal Ethical and Welfare Committee (IACUC-20181015-05) of Zhejiang Chinese Medical University. All animal studies were conducted in strict accordance with the guidelines of the Animal Care and Use Committee of Animal Ethical and Welfare Committee of Zhejiang Chinese Medical University. Twenty-four 6-week-old NOD/SCID mice (SHANGHAI SLAC, Shanghai) were purchased and randomly divided into four groups. The mice were raised under specific pathogen-free (SPF), 12-hour light and dark cycle conditions, with a room temperature of 22 ± 0.5 °C, a relative humidity of 40%–70%, and freely access to feed and water. PANC-1 cells were infected with lentiviruses harboring an MYEOV overexpression vector [PANC-1 (MYEOV)] or its negative control blank vector (PANC-1 (NEG)] or with an MYEOV shRNA knockdown vector [PANC-1 (shMYEOV)] or its negative control blank vector [PANC-1 (shNEG)]. Stable cell lines were obtained by screening with puromycin. To evaluate cell proliferation in vivo, the four stable cell lines described above were subcutaneously injected into the four groups of mice (8 × 10^6^ per mouse). The mice were monitored weekly for tumor growth and size and were sacrificed 8 weeks post-injection. Before the mice were sacrificed, they were euthanized with carbon dioxide inhalation to alleviate pain. All the tumors were harvested and weighed for analysis. Tumor growth was measured weekly with a caliper from weeks 1 to 8. Tumor volume analyses were based on the following equation: V = (length × width^2^)/2.

### Chromatin immunoprecipitation (ChIP)

ChIP was conducted in PANC-1 cells using a SimpleChIP Enzymatic Chromatin IP kit (#9002; Cell Signaling Technology, USA) according to the manufacturer’s protocol. Cells were fixed with 1% formaldehyde for 10 min at room temperature, and fixation was stopped by adding glycine followed by an additional incubation at room temperature for 5 min. Subsequently, the cells were scraped, pelleted and then lysed in 1 mL of Buffer A supplemented with a protease inhibitors cocktail (PIC) and DTT for 10 min on ice. After centrifugation at 2,000 × g for 5 min at 4 °C, the pelleted nuclei were resuspended in 1 mL of Buffer B supplemented with DTT and incubated for 10 min at 4 °C. After repeating the centrifugation step, the pelleted nuclei were resuspended in 100 µl of Buffer B, mixed with 0.5 µl of micrococcal nuclease, and then incubated for 20 min at 37 °C with frequent mixing to digest the DNA into approximately 150- to 900-bp fragments. Next, 10 µl of 0.5 M EDTA was added to stop the digestion. After centrifugation at 16,000 × g for 1 min at 4 °C, the pelleted nuclei were resuspended in 500 µl of ChIP buffer and sonicated for 20 min. After centrifugation at 9,400 × g for 10 min at 4 °C, the supernatant was obtained as the cross-linked chromatin preparation. For optimal ChIP results, we used approximately 5-10 µg of digested, cross-linked chromatin per IP reaction. For each IP, we added the immunoprecipitating antibodies to 500 µl of the diluted chromatin. After incubating the IP samples 4 h to overnight at 4 °C with rotation, ChIP-Grade Protein G Agarose Beads were added to each IP reaction. After a 2-h incubation at 4 °C with rotation, the beads were washed three times with 1 mL of low-salt wash buffer and then once with 1 mL of high-salt wash buffer. The DNA was eluted in elution buffer, and crosslinks were reversed by incubation overnight at 65 °C. The RNA and protein were digested using RNase A and Proteinase K, and the DNA was purified by phenol-chloroform extraction and isopropanol precipitation. The target DNA abundance in ChIP eluates was assayed by qPCR with primer pairs designed to generate 100- to 200-bp products. An isotype-matched IgG antibody was used as a control. The antibodies used for ChIP assays were as follows: anti-MYC (#9402; Cell Signaling Technology), anti-Histone H3 (D2B12) XP® Rabbit mAb (#4620; Cell Signaling Technology), and normal rabbit IgG (#2729; Cell Signaling Technology). The sequences of the primers used for ChIP assays were as follows: positive B23 gene forward primer: 5’-GCTACATCCGGGACTCACC-3’; positive B23 gene reverse primer: 5’-GCTGCCATCACAGTACATGC-3’; miR-17 gene cluster promoter forward primer: 5’-AAAGGCAGGCTCGTCGTTG-3’; miR-17 gene cluster promoter reverse primer: 5’-CGGGATAAAGAGTTGTTTCTCCAA-3’; miR-93 gene promoter forward primer: 5’-ATTGGCCATCACACCCAGAG-3’; and miR-93 gene promoter reverse primer: 5’-GTGTAAACAGTGTCCTTCCGC-3’.

### Bioinformatics analysis

The GEPIA (Gene Expression Profiling Interactive Analysis) webserver [34], which integrates TCGA and GTEx data, was accessed to perform MYEOV-related differential gene and survival analyses using the “Differential Genes Analysis” and “Most Significant Survival Genes” modules, respectively. All available samples classified as PDAC (*n* = 178) in TCGA and non-cancerous pancreatic (NCP) tissue samples (*n* = 171) in TCGA and GTEx were used to perform differential analysis based on the MYEOV expression profiles. The clinicopathological features and MYEOV gene expression values (measured as fragments per kilobase million, FPKM) corresponding to these samples were extracted from the TCGA and GETx resource centers. The receiver operating characteristic (ROC) curve based on MYEOV abundance was plotted, and the area under the ROC curve (AUC) was calculated using R (v 3.5.1).

Survival analysis in GEPIA was first conducted via the “Most Differential Survival Genes” module to identify genes associated with PDAC patient survival using the following parameters: Datasets Selection = PAAD, Methods = Overall Survival, and Group Cutoff = Median. Next, the “Survival Plots” module was used to generate survival curves corresponding to specific genes based on expression levels using the following parameters: Gene = MYEOV, Methods = Overall Survival or Disease Free Survival, Group Cutoff = Median, Hazards Ratio (HR) = Yes, 95% Confidence Interval = Yes, Axis Units = Months, and Datasets Selection = PAAD.

### RNA-Seq experiments

The RNA sequencing experiments and downstream data analysis were performed as described by Hu et al. [35]. Briefly, total RNA from each sample was extracted using TRIzol reagent (Invitrogen) and used to prepare a cDNA library based on the standard Illumina RNA-Seq protocol. The generated cDNA library was sequenced on an Illumina HiSeq2000 instrument in a 2 × 150 bp layout. To estimate gene expression abundance and differentially expressed genes (DEGs) among the samples, the obtained raw RNA-Seq datasets were filtered using Trimmomatic to remove low-quality reads and potential adapter contamination [36]. The quality-controlled reads were then mapped to the human genome (hg19) using HISAT2 with default parameters [37]. Only uniquely mapped reads were retained to quantify gene expression abundance at the count level based on the Ensembl human gene annotation [38]. The DEGs with significant differences and log2-transformed gene expression fold changes (LFCs) were estimated using edgeR after TMM normalization (FDR < 0.05) [39]. All the original RNA-Seq data were deposited in the GEO database (GEO accession number: GSE143828).

### Small RNA sequencing experiments

Cells were harvested after 24 h, and total RNA was extracted using TRIzol reagent (Invitrogen) to prepare a small RNA library based on the standard Illumina small RNA sequencing protocol. The generated library was sequenced on an Illumina HiSeq2500 instrument in a 1 × 50 bp layout. We mapped the obtained small RNA sequencing data following the mapping steps according to the method described by Hu [40]. To remove the adapter sequence at the 3’ end of the raw sequence reads, all the sequences were trimmed using a custom trimming procedure [41]. The trimmed sequences were then mapped to the human genome (hg19) using Bowtie [42]. Only sequences that perfectly matched the genome and had a length ranging from 18 to 28 nucleotides were retained for miRNA expression quantification. We quantified miRNA expression abundance following the quantification method described by Hu [43]. First, we collected all the sequences that mapped within three nucleotides upstream or downstream of the 5’ end position of each mature miRNA. Next, for each mature miRNA, the sequence with the maximal expression count number was designated as the reference sequence. The expression abundance of each miRNA was further estimated as the sum of the count number of the reference sequence and sequences mapping at the same 5’ end position as the reference sequence.

### Statistical analysis

All experiments were independently performed at least three times. The values are presented as means ± standard error of the mean (SEM). Differences between two groups and more than two groups were assessed by two-tailed Student’s t-test and one-way analysis of variance (ANOVA), respectively. Analysis was performed using Microsoft Excel 2017, SPSS 22.0 and GraphPad Prism (Prism 5 for Windows). A P value of less than 0.05 indicated statistical significance.

## Results

### MYEOV overexpression is associated with poor patient survival in PDAC

**Fig. 1:**
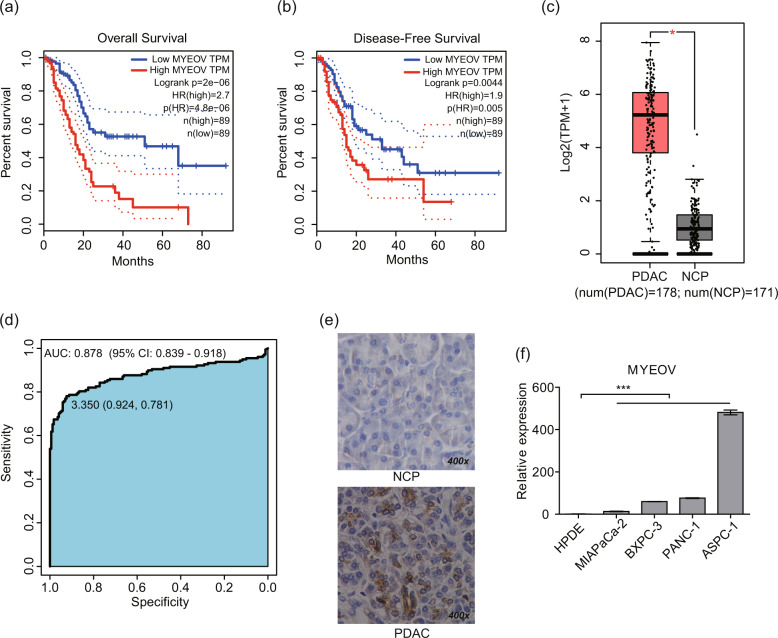
MYEOV is correlated with the poor prognosis of PDAC patients and is highly expressed in PDAC cells. **a**, **b** Kaplan–Meier curves of overall survival (**a**) and disease-free survival (**b**) of patients with PDAC based on MYEOV expression in the GEPIA database using the log-rank test. The dotted lines indicate the 95% confidence intervals for survival percentage at each time point as obtained by survival analysis. **c** MYEOV expression levels in PDAC (*n* = 178) and NCP tissues (*n* = 171) based on the expression levels in GEPIA database. Gene expression profiles of MYEOV from all available samples classified as PDAC tissue (*n* = 178) in TCGA and NCP tissue (*n* = 171) in TCGA and GTEx were chosen to perform differential analysis in GEPIA. The data are presented as the means ± SEM. *P < 0.05 (one-way ANOVA). **d** ROC curves based on the gene expression profiles of MYEOV in PDAC and normal tissue samples. The AUC value with a 95% CI is indicated. **e** Representative images of the IHC staining analyses of 19 FFPE PDAC and NCP tissues using an anti-MYEOV antibody (400× magnification). **f** Comparison of MYEOV expression levels in PDAC cell lines and normal pancreatic epithelial cells by qPCR. All *n* ≥ 3; bar, SEM; ***P < 0.001; Student’s *t*-test. ANOVA, analysis of variance; PDAC, Pancreatic ductal adenocarcinoma; NCP, non-cancerous pancreas; TPM, transcripts per kilobase million; ROC, receiver operating characteristic.

To identify the genes associated with PDAC survival, we performed survival analysis based on TCGA [44] and GTEx [45, 46] data integrated in GEPIA (Table S2). A higher MYEOV level in PDAC was correlated with a worse overall survival and disease-free survival outcomes (Fig. 1a, b). Additionally, the expression levels of MYEOV were clearly increased in PDAC tissues (*n* = 178) compared with NCP tissues (*n* = 171) (*P* < 0.05, Fig. 1c). Based on the expression profiles of MYEOV in the PDAC and NCP tissue samples, the AUC was determined as 0.878 (95% confidence interval (CI): 0.839-0.918; Fig. 1d), indicating that this aberrantly expressed gene may be of great diagnostic value to distinguish PDAC from NCP tissues. Furthermore, we explored the correlation between the MYEOV expression levels and clinicopathological features in 178 PDAC patients (Table 1). To validate the results of the in silico studies, in situ hybridization (ISH) staining was performed, the results of which confirmed higher MYEOV expression in 19 FFPE PDAC samples than in the matched adjacent normal tissues (Fig. 1e, S1a, b). Consistently, the prognosis of patients with high expression of MYEOV was significantly worse than that of patients with low expression of MYEOV (*P* < 0.05) (Fig. S1c). After observing increased MYEOV expression in PDAC, we analyzed MYEOV expression in 4 PDAC cell lines and 1 normal HPDE cell line and confirmed the increased MYEOV expression levels in cancer cells (Fig. 1f; *P* < 0.05). Overall, these findings indicate that survival-related MYEOV is overexpressed in PDAC.

**Table 1:** Correlation of MYEOV expression with clinicopathological features in PDAC samples from the TCGA database.

Clinicopathological features	*n*	Mean expression of MYEOV (FPKM)	*P* value
**Gender**			
Male	98	17.25	0.189
Female	80	14.14	
**TNM stage**			
I	21	9.8	0.084
II	146	17.34	
III	3	14.28	
IV	5	5.72	
Not reported	3	4.42	
**Race**			
Asian	11	16.25	0.487
Black or African American	6	6.02	
White	157	16.21	
Not reported	4	15.46	

### MYEOV deficiency leads to reduced proliferation, invasion and migration of PDAC cells in vitro and in vivo

**Fig. 2:**
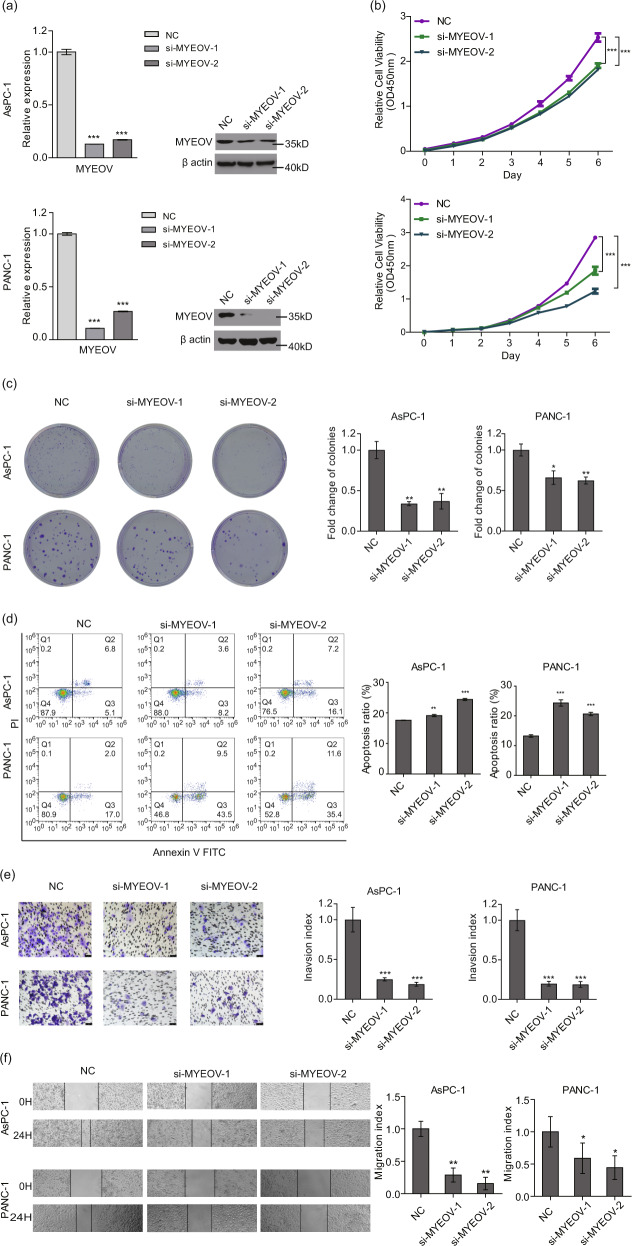
MYEOV knockdown inhibits cell proliferation, invasion and migration in PDAC in vitro. **a** The expression levels of MYEOV were quantified by qPCR and Western blot. **b**, **c** Cell proliferation measured by the CCK-8 (**b**) and colony formation assays (**c**). **d** Apoptosis as determined by flow cytometry. **e**, **f** Cell invasion (**e**) and migration (**f**) as determined by transwell invasion assays (200× magnification) and scratch assays (40× magnification). All *n* ≥ 3; bar, SEM; *P < 0.05, **P < 0.01, ***P < 0.001 and ****P < 0.0001 compared with NC; Student’s *t*-test.

To assess the role of MYEOV in PDAC cell proliferation, we depleted MYEOV in two PDAC cancer cell lines (AsPC-1 and PANC-1) by transfecting cells with MYEOV-targeting siRNAs and then performing CCK-8 and colony formation assays. The viability of MYEOV-knockdown cells was significantly reduced compared with that of the corresponding control cells (Fig. 2a, b, c). Additionally, to validate the outcomes of MYEOV depletion assays, we overexpressed MYEOV in PANC cells and observed increased cell viability (Fig. S2a). Subsequently, we investigated the role of MYEOV in cell apoptosis using flow cytometry. The apoptosis rates were elevated after MYEOV depletion (Fig. 2d). To further examine the influence of MYEOV on the migration and invasion of PDAC cells, wound healing and Matrigel Transwell assays were performed. MYEOV-depleted cells had lower recovery rates and reduced cell invasion ability than the control cells (Fig. 2e, f). These in vitro results demonstrate that MYEOV likely plays a pro-cancer biological role in PDAC cells by promoting cell proliferation, migration and invasion.

**Fig. 3:**
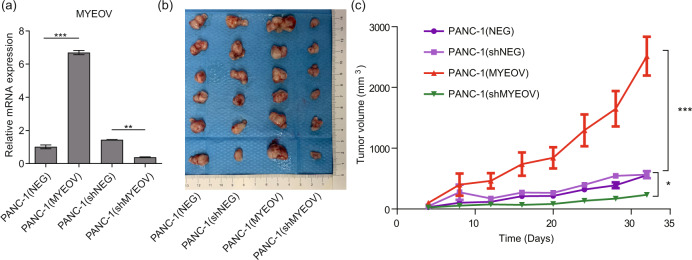
MYEOV knockdown inhibits cell growth in PDAC in vivo. **a** The qPCR-based expression levels of MYEOV in four types of stable PANC-1 cell lines. PANC-1 cells were infected with lentiviruses harboring a MYEOV overexpression vector [PANC-1 (MYEOV)] or its negative control blank vector [PANC-1 (NEG)] or with a MYEOV shRNA knockdown vector [PANC-1 (shMYEOV)] or its control negative blank vector [PANC-1 (shNEG)]. **b**, **c** Tumor volume (**b**) and tumor growth curves (**c**) after four types of stable cell lines were injected into NOD/SCID mice. All *n* ≥ 3; bar, SEM; *P < 0.05, **P < 0.01 and ***P < 0.001 compared with NC; Student’s *t*-test.

Xenograft mouse models were used to determine the in vivo functions of MYEOV. The qPCR results validated MYEOV expression in the four types of PANC-1 cells, with increased levels observed in tumors derived from mice injected with MYEOV overexpression cells and decreased levels in tumors derived from MYEOV-knockdown cells compared with the controls (Fig. 3a). Consistent with the in vitro findings, significant decreases in the tumor volume and growth rates were observed in mice injected with MYEOV-knockdown PANC-1 cells compared with those in the negative control group, whereas mice injected with MYEOV-overexpressing cells showed the opposite effect (Fig. 3b, c). Taken together, our data demonstrated that MYEOV deficiency leads to reduced proliferation of PDAC cells in vitro and in vivo.

### Global effect of MYEOV depletion on the transcriptome in PDAC cells

**Fig. 4:**
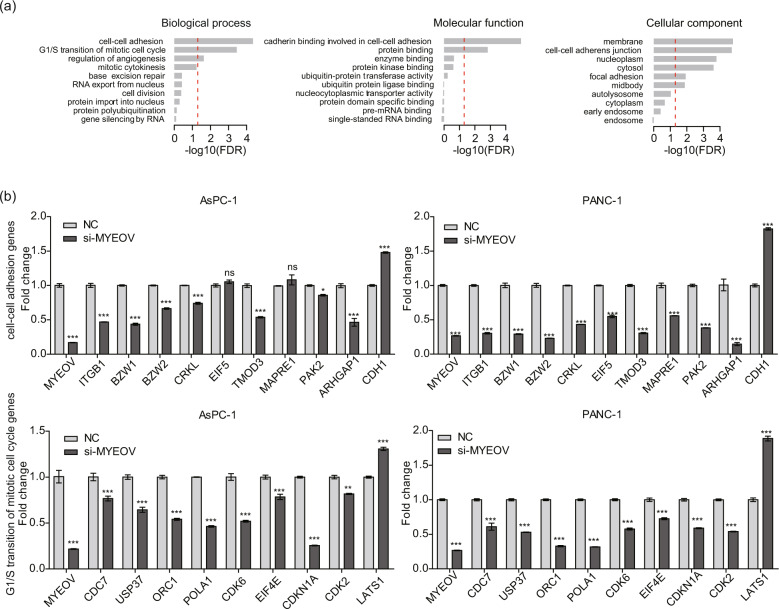
Global effect of MYEOV depletion on the transcriptome in PDAC cells. **a** GO enrichment analysis for differentially regulated mRNAs in MYEOV-depleted AsPC-1 and PANC-1 cells. The red dashed line indicates an FDR cut-off of value of 0.05. The *y*-axis represents specific GO items of three GO categories (biological process, molecular function and cellular component). The *x*-axis represents the enrichment magnitude of a specific GO item as measured by the FDR of Fisher’s exact test on a -log10 scale. **b** qPCR validation of selected genes during the cell-cell adhesion and G1/S transition of the mitotic cell cycle processes in PDAC cells. All *n* = 3; bar, SEM; *P < 0.05, **P < 0.01 and ***P < 0.001 compared with NC; Student’s *t*-test.

Having demonstrated that MYEOV can promote tumor progression, we further investigated MYEOV-mediated changes in the global transcriptome in MYEOV-depleted PDAC cells (PANC-1 and AsPC-1) by mRNA-Seq. MYEOV depletion resulted in the differential expression of 1005 genes (negative binomial test; FDR < 0.05), with 409 genes upregulated and 596 downregulated, including MYEOV itself (Fig. S2b). Gene ontology (GO) analysis of DEGs following MYEOV knockdown revealed an enrichment of genes involved in cell-cell adhesion, G1/S transition of the mitotic cell cycle, regulation of angiogenesis and other cellular processes (Fig. 4a). These DEGs were primarily enriched in molecular functions, such as cadherin binding and protein binding. Furthermore, the DEG profile identified by GO analysis was further cross-validated by analyzing the expression levels of these genes via qPCR (Fig. 4b, Table S3). Except for the differential expression of the two genes EIF5 and MAPRE1, which were not validated in AsPC-1 cells, the remaining qPCR results were consistent with mRNA-Seq. Collectively, these results suggest that MYEOV is a crucial oncogene that causes global alterations in the gene expression profiles of PDAC cells.

### MYEOV-regulated miR-17/93-5p affects PDAC cell proliferation, invasion and migration

**Fig. 5:**
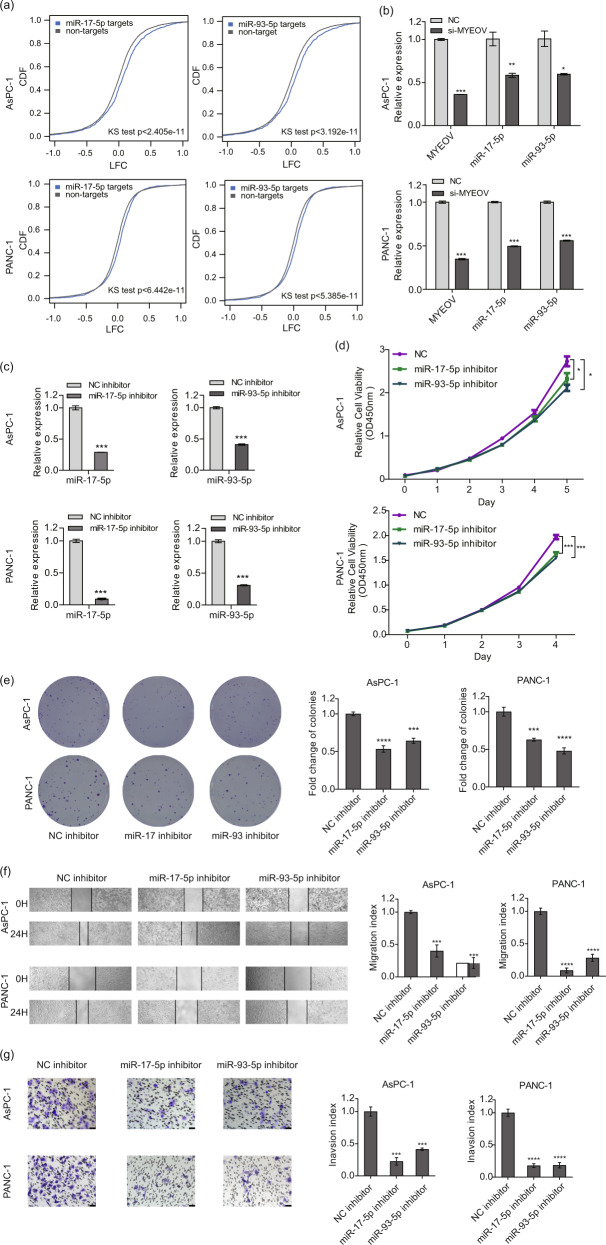
Downregulation of miR-17-5p and miR-93-5p produces a similar effect as that observed with MYEOV depletion in PDAC cells. **a** Changes in the expression of miR-17-5p and miR-93-5p target genes after MYEOV knockdown in two PDAC cell lines (AsPC-1 and PANC-1). The x-axis represents the LFC between MYEOV-knockdown and wild-type PDAC cells. The y-axis represents the cumulative distribution function (CDF) of LFC distribution. In each comparison, all expressed genes (excluding the target genes of cognate miRNAs) were used as the background. The cumulative distribution difference between targets and background genes was estimated using the Kolmogorov-Smirnov test. **b** The qPCR-based expression levels of miR-17-5p and miR-93-5p upon MYEOV knockdown. **c** The qPCR-based expression levels of miR-17-5p and miR-93-5p upon treatment with miRNA inhibitors. **d**, **e** Cell proliferation as measured by the CCK-8 (**d**) and colony formation (**e**) assays in AsPC-1 and PANC-1 cell lines transfected with the miR-17-5p inhibitor, miR-93-5p inhibitor or NC. **f**, **g** Cell migration (**f**) and invasion (**g**) as determined by scratch assay (40× magnification) and transwell invasion assay (200× magnification), respectively. All *n* = 3; bar, SEM; *P < 0.05, **P < 0.01 and ***P < 0.001 compared with NC; Student’s *t*-test.

As important gene regulators, miRNAs are involved in the development and progression of PDAC. To identify the miRNAs regulated by MYEOV, we performed high-throughput miRNA-Seq in MYEOV-depleted PDAC cells (PANC-1 and AsPC-1). We identified 54 and 39 differentially expressed (DE) miRNAs after MYEOV depletion in PANC-1 and AsPC-1 cells, respectively (Fig. S2c; negative binomial test; FDR < 0.05). Fifteen miRNAs displayed differential expression in both PDAC cancer cell lines that was significantly more than expected by chance (hypergeometric test; *P* < 0.05). Moreover, the changes in expression of these 15 DE miRNAs were positively correlated between PANC-1 and AsPC-1 cells (Fig. S2d; Pearson’s correlation coefficient α = 0.85; *P* < 0.0001). These results indicated that MYEOV depletion disrupted the miRNA transcription machinery and led to aberrantly expressed miRNAs in both PDAC cells. To further pinpoint the most relevant DE miRNAs, we examined the regulatory effect of each of these 15 DE miRNAs on the expression of their cognate target genes. Because miRNAs are typically negative regulators, we calculated whether the up-/downregulation of miRNAs led to significantly reversed expression changes in their target genes by considering all the expressed genes as the background. We detected significant regulatory effects of two miRNAs, miR-17-5p and miR-93-5p, on their cognate target genes in both PDAC cells (Fig. 5a; Kolmogorov**-**Smirnov test; all *P* < 1e-10), whereas no regulatory effect signal was observed for the remaining DE miRNAs (Kolmogorov**-**Smirnov test; all *P* > 0.05), suggesting miR-17-5p and miR-93-5p were the most relevant miRNAs associated with MYEOV-induced gene expression changes. Subsequently, the qPCR results verified that MYEOV knockdown resulted in miR-17-5p and miR-93-5p downregulation in PDAC cells (Fig. 5b). To investigate the biological functions of miR-17-5p and miR-93-5p in PDAC cells, endogenous miR-17-5p or miR-93-5p was knocked down in PANC-1 and AsPC-1 cells with a specific miRNA inhibitor (Fig. 5c). We observed that the depletion of either miR-17-5p or miR-93-5p significantly inhibited cell proliferation, invasion and migration (Fig. 5d, e, f, g), all of which were also observed in MYEOV-depleted PDAC cells. Additionally, miR-17-5p or miR-93-5p inhibition did not affect MYEOV expression (Fig. S2e). These results suggest that the inhibition of miR-17-5p or miR-93-5p negatively affects PDAC cell growth and has a similar effect on PDAC progression to that observed in MYEOV-depleted PDAC cells.

**Fig. 6:**
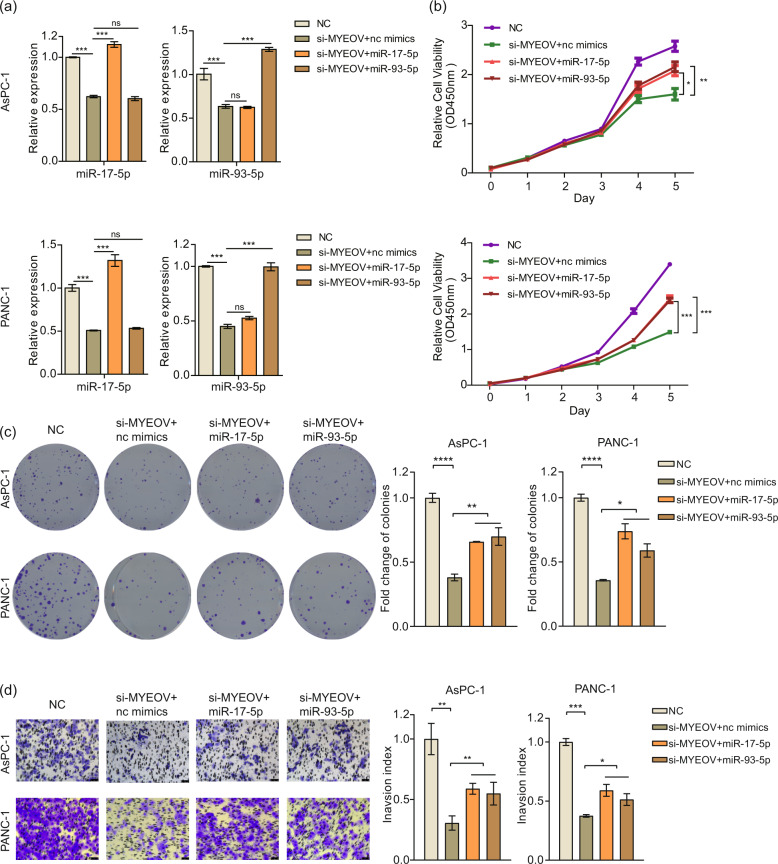
Exogenous miR-17-5p and miR-93-5p expression partially reverses the reduction in cell proliferation, migration and invasion caused by MYEOV knockdown. **a** The qPCR-based expression levels of miR-17-5p and miR-93-5p before and after transfection. **b**, **c** Cell proliferation as measured by the CCK-8 (**b**) and colony formation (**c**) assays. **d** Cell invasion as determined by the transwell invasion assay (200× magnification). All *n* ≥ 3; bar, SEM; *P < 0.05, **P < 0.01 and ***P < 0.001; Student’s *t*-test.

To investigate the functional relevance of MYEOV with miR-17-5p or miR-93-5p, we assessed whether miR-17-5p or miR-93-5p overexpression neutralizes the inhibitory effects of MYEOV deficiency on PANC-1 cell proliferation, migration and invasion. qPCR was used to validate the levels of miR-17-5p or miR-93-5p in the rescue experiment (Fig. 6a). Exogenous miR-17-5p or miR-93-5p expression partially reversed the reduced cell proliferation, metastasis and invasion due to MYEOV knockdown in PDAC cells (Fig. 6b, c, d). To investigate whether miR-17/93-5p served as a downstream molecule of MYEOV, we overexpressed MYEOV in miR-17/93-5p-inhibitory PDAC cells and assessed the alteration in cell proliferation. qPCR confirmed that the expression levels of MYEOV, miR-17-5p or miR-93-5p were consistent as expected (Fig. S3a). MYEOV overexpression could not reverse the reduction in cell proliferation caused by miR-17/93-5p knockdown in PDAC cells (Fig. S3b), indicating that miR-17/93-5p might serve as a downstream target of MYEOV. Thus, MYEOV influences PDAC cells partially by regulating miR-17/93-5p expression.

### MYC is involved in the regulation of MYEOV on miR-17/93-5p

**Fig. 7:**
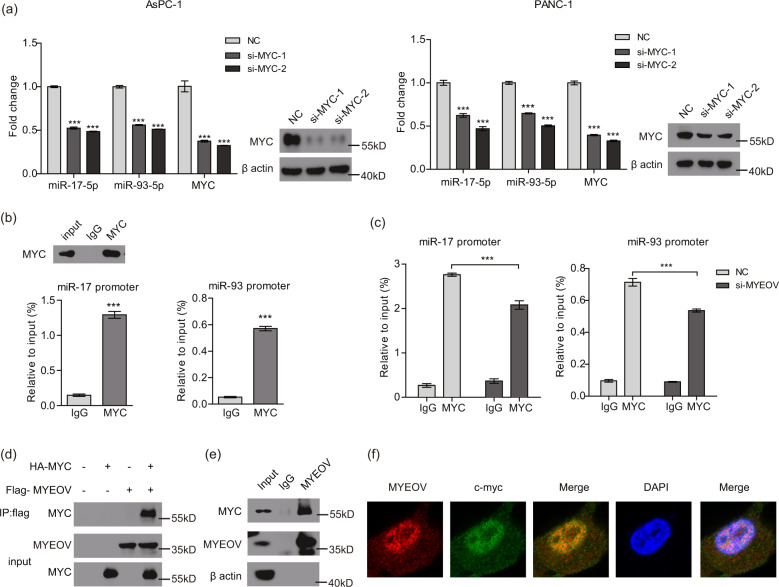
MYC is involved in the MYEOV-mediated regulation of miR-17/93-5p. **a** The qPCR-based expression levels of miR-17-5p and miR-93-5p upon MYC knockdown. **b** ChIP analysis of MYC enrichment at the promoters of miR-17 and miR-93 genes in PANC-1 cells. Western blotting was performed to detect MYC protein after immunoprecipitation, where IgG was used as a negative control. An isotype-matched IgG antibody was used as a control in ChIP analysis. **c** ChIP analysis of MYC enrichment at the promoters of the miR-17 and miR-93 genes in control and MYEOV-silenced PANC-1 cells. ChIP values are expressed as the means ± SEM of relative input chromatin (%). An isotype-matched IgG antibody was used as a control. **d** Association of FLAG-tagged MYEOV with HA-tagged MYC as detected by immunoprecipitation. **e** Endogenous interaction between MYEOV and MYC as revealed by co-immunoprecipitation. **f** Endogenous interaction between MYEOV and MYC as detected by immunofluorescence. MYEOV (Red); MYC (Green); Nucleus (Blue). All *n* = 3; bar, SEM; *P < 0.05, **P < 0.01 and ***P < 0.001; Student’s *t*-test.

We subsequently investigated how MYEOV regulates the expression levels of miR-17-5p and miR-93-5p. According to previous reports, the transcription factor MYC can directly upregulate miR-17-5p expression and is associated with miR-93-5p expression [47–49]. The promoter regions of both miR-17 and miR-93 genes contain canonical sequences for MYC. As expected, MYC expression is high in PDAC cells compared with that in normal pancreatic epithelial cells (Fig. S2f). After MYC knockdown, the levels of miR-17-5p and miR-93-5p were notably decreased (Fig. 7a). Because the oncogenic potential of MYC stems from its function as a transcriptional regulator that binds to DNA with an active chromatin configuration, we wondered whether MYC binds to the gene promoter regions of miR-17/93. By conducting ChIP experiments using an anti-MYC antibody, we observed a significant and specific enrichment of MYC at the promoter regions of miR-17 and miR-93 (Fig. 7b). Subsequently, we performed ChIP-qPCR to determine whether MYEOV silencing interfered with MYC recruitment to the promoter regions of miR-17 and miR-93. Notably, the recruitment of MYC to miR-17/93 promoters was markedly reduced in MYEOV-depleted cells (Fig. 7c). These results preliminarily demonstrated that MYEOV regulates miR-17/93 expression by affecting the enrichment of MYC at the promoter regions of these two miRNAs.

To activate gene transcription, MYC typically recruits chromatin-modifying and chromatin-remodeling complexes to increase DNA accessibility at the target region. Therefore, we speculated that the association of MYEOV with MYC might mechanistically explain the enhancement of MYC transcriptional regulation on miR-17/93-5p. IP of FLAG-MYEOV coupled with Western blotting was performed to detect HA-MYC from the lysates of transfected 293 T cells, with the results revealing that both proteins were present in the same complex (Fig. 7d) and that the interaction between them was dose dependent (Fig. S2g). Furthermore, the association between endogenous MYC and MYEOV was validated in PANC-1 cells through co-IP and immunofluorescence analyses (Fig. 7e, f). Taken together, these data indicate that MYEOV interacts with MYC in the cell nucleus to regulate the expression of downstream miR-17/93-5p.

## Discussion

**Fig. 8:**
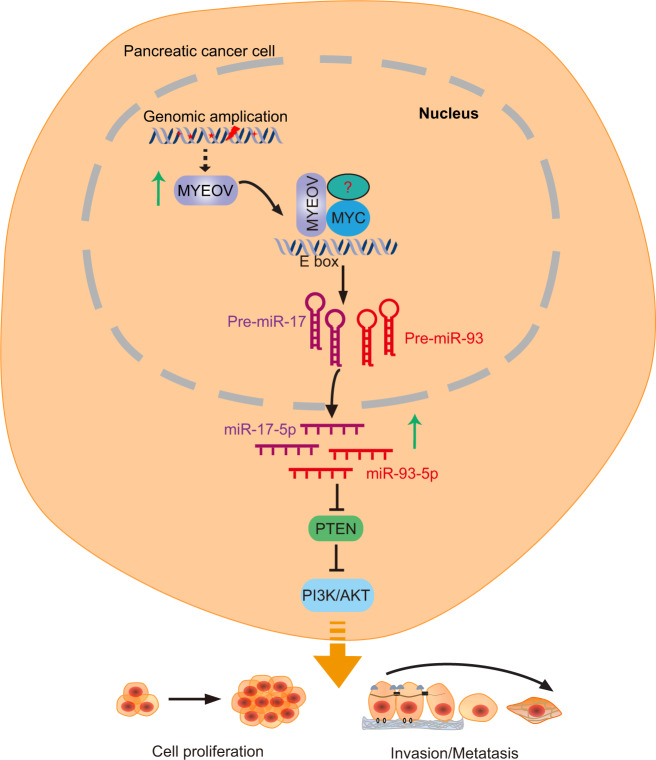
Proposed model for MYEOV role in PDAC is schematized. MYEOV can potentiate PDAC proliferation, invasion and metastasis by up-regulating the expression of miR-17/93-5p via its association with the transcription factor MYC. MiR-17/93-5p might then regulate the PI3K/Akt pathway via inhibiting the expression of PTEN.

A key finding of our study is that MYEOV can potentiate PDAC proliferation, invasion and metastasis by regulating the expression of miR-17/93-5p via its association with the transcription factor MYC (Fig. 8). This finding extends our current understanding of the molecular mechanism mediating PDAC progression.

Our results showed that MYEOV is associated with poor survival in patients and is overexpressed in PDAC. MYEOV deficiency led to a reduction in PDAC cell proliferation, invasion and migration. Depletion of MYEOV significantly affected tumor formation induced by PDAC cells in a xenograft mouse model. These in vitro and in vivo results demonstrated that MYEOV contributes to tumorigenesis in PDAC. This is consistent with the role of MYEOV in NSCLC, which has been reported by Fang et al. [14]. However, according to a report by Fang, since no MYEOV protein expression is observed in NSCLC cells and tissues, MYEOV functions as an amplified competing endogenous RNA (ceRNA) to enhance NSCLC metastasis. In our study, we observed high MYEOV protein expression in PDAC cells and tissues, which may be due to the specific expression patterns of MYEOV in different tissues and organs. However, we cannot exclude the possibility that MYEOV may also play a role as a ceRNA in PDAC.

Accumulating evidence shows that miRNAs are involved in the carcinogenesis and metastasis of various human cancers, including PDAC [50, 51]. In this study, through high-throughput miRNA-Seq, we showed that MYEOV affects the expression of miR-17-5p and miR-93-5p. MiR-17-5p and miR-93-5p, the most representative miRNAs with pro-cancer properties, have been reported to play an oncogenic role in various cancers [31, 52, 53]. PTEN, which is the key downstream target of miR-17-5p and miR-93-5p, is potentially involved in the regulation of cell viability and apoptosis and it negatively regulates the PI3K/Akt pathway [54–57]. Therefore, we hypothesized that MYEOV might be able to regulate PI3K/Akt signaling pathway by targeting PTEN. In our study, we observed that downregulation of either miR-17-5p or miR-93-5p exhibited a similar effect as that observed with MYEOV depletion in human PDAC cells. Furthermore, miR-17-5p or miR-93-5p overexpression could rescue the inhibitory effects of MYEOV deficiency in PDAC cells. These data allowed us to conclude that MYEOV partially influences the proliferation of PDAC cells by regulating the expression of miR-17-5p and miR-93-5p. Indeed, it has been well recognized that miR-17/93-5p can regulate a cohort of target genes involved in various biological processes [58]. Given that the biological functions of MYEOV-depleted PDAC cells were partially restored after exogenous miR-17-5p and miR-93-5p expression, it is possible that MYEOV exerts its function by targeting genes other than miR-17/93-5p. Thus, the oncogenic role of MYEOV in PDAC requires further investigation.

The oncogene MYC has been implicated in the pathogenesis of various malignancies, including many PDAC cases. Characterized as a driver of tumor biological functions, MYC overexpression frequently predicts a poor clinical outcome and an aggressive course of disease [59]. MYC has been reported to control the expression of miRNAs, and MYC-regulated miRNAs affect virtually all aspects of the hallmarks of MYC-driven pathology. In our study, we observed that MYC associated with MYEOV by interacting in a complex to regulate downstream miR-17/93-5p expression in PDAC. We cannot rule out the possibility that MYC may also regulate the target genes involved in the pro-cancer function of MYEOV. MiR-17-5p has been reported to counterbalance MYC expression and function to ensure optimal B cell lymphoma growth [60]. In our results, miR-17/93-5p overexpression in PDAC cells had a slight effect on the expression of MYEOV and MYC, which indicated that there exists potential synergism between MYC and miR-17/93-5p in PDAC. Additionally, the oncogenic potential of MYC stems from its function as a transcriptional factor that interacts with a diverse array of factors to bind DNA [23]. Mounting evidence indicates that forming complexes with different factors leads to different regulatory modes for the expression of downstream genes [23, 61], and whether there are other factors involved in the MYC and MYEOV complex needs to be further elucidated.

## Conclusions

In summary, the results of our present study demonstrated a previously undiscovered role of MYEOV in PDAC, as we showed that elevated MYEOV expression promotes PDAC growth. Further molecular analysis revealed that MYEOV acts as a tumor promoter by associating with MYC to facilitate oncogenic miR-17/93-5p expression in PDAC progression and may provide a potential therapeutic strategy for PDAC patients.

## Supplementary information

Fig. S1-3

Table S1

Table S2

Table S3

## Data Availability

All data generated or analyzed during this study are included in this published article.

## References

[1] Balachandran VP, Luksza M, Zhao JN, Makarov V, Moral JA, Remark R (2017). Identification of unique neoantigen qualities in long-term survivors of pancreatic cancer. Nature.

[2] Von Hoff DD, Korn R, Mousses S (2009). Pancreatic cancer-could it be that simple? A different context of vulnerability. Cancer Cell.

[3] Rachagani S, Macha MA, Heimann N, Seshacharyulu P, Haridas D, Chugh S (2015). Clinical implications of miRNAs in the pathogenesis, diagnosis and therapy of pancreatic cancer. Adv Drug Deliv Rev.

[4] Srivastava SK, Arora S, Singh S, Bhardwaj A, Averett C, Singh AP (2014). MicroRNAs in pancreatic malignancy: progress and promises. Cancer Lett.

[5] Srivastava SK, Bhardwaj A, Singh S, Arora S, Wang B, Grizzle WE (2011). MicroRNA-150 directly targets MUC4 and suppresses growth and malignant behavior of pancreatic cancer cells. Carcinogenesis.

[6] Janssen JW, Vaandrager JW, Heuser T, Jauch A, Kluin PM, Geelen E (2000). Concurrent activation of a novel putative transforming gene, myeov, and cyclin D1 in a subset of multiple myeloma cell lines with t(11;14)(q13;q32). Blood.

[7] Hui AB, Or YY, Takano H, Tsang RK, To KF, Guan XY (2005). Array-based comparative genomic hybridization analysis identified cyclin D1 as a target oncogene at 11q13.3 in nasopharyngeal carcinoma. Cancer Res.

[8] Brown LA, Irving J, Parker R, Kim H, Press JZ, Longacre TA (2006). Amplification of EMSY, a novel oncogene on 11q13, in high grade ovarian surface epithelial carcinomas. Gynecologic Oncol.

[9] Rodrigo JP, Garcia-Carracedo D, Garcia LA, Menendez S, Allonca E, Gonzalez MV (2009). Distinctive clinicopathological associations of amplification of the cortactin gene at 11q13 in head and neck squamous cell carcinomas. J Pathol.

[10] Janssen JW, Cuny M, Orsetti B, Rodriguez C, Valles H, Bartram CR (2002). MYEOV: a candidate gene for DNA amplification events occurring centromeric to CCND1 in breast cancer. Int J cancer.

[11] Leyden J, Murray D, Moss A, Arumuguma M, Doyle E, McEntee G (2006). Net1 and Myeov: computationally identified mediators of gastric cancer. Br J Cancer.

[12] Moss AC, Lawlor G, Murray D, Tighe D, Madden SF, Mulligan AM (2006). ETV4 and Myeov knockdown impairs colon cancer cell line proliferation and invasion. Biochem Biophys Res Commun.

[13] Moreaux J, Hose D, Bonnefond A, Reme T, Robert N, Goldschmidt H (2010). MYEOV is a prognostic factor in multiple myeloma. Exp Hematol.

[14] Fang L, Wu S, Zhu X, Cai J, Wu J, He Z (2019). MYEOV functions as an amplified competing endogenous RNA in promoting metastasis by activating TGF-beta pathway in NSCLC. Oncogene.

[15] Bartel DP (2004). MicroRNAs: genomics, biogenesis, mechanism, and function. Cell.

[16] Rupaimoole R, Slack FJ (2017). MicroRNA therapeutics: towards a new era for the management of cancer and other diseases. Nat Rev Drug Discov.

[17] Manikandan M, Deva Magendhra Rao AK, Arunkumar G, Manickavasagam M, Rajkumar KS, Rajaraman R (2016). Oral squamous cell carcinoma: microRNA expression profiling and integrative analyses for elucidation of tumourigenesis mechanism. Mol Cancer.

[18] Ottaviani S, Stebbing J, Frampton AE, Zagorac S, Krell J, de Giorgio A (2018). TGF-beta induces miR-100 and miR-125b but blocks let-7a through LIN28B controlling PDAC progression. Nat Commun.

[19] Yuan P, He XH, Rong YF, Cao J, Li Y, Hu YP (2017). KRAS/NF-kappaB/YY1/miR-489 signaling axis controls pancreatic cancer metastasis. Cancer Res.

[20] Dang CV, O’Donnell KA, Zeller KI, Nguyen T, Osthus RC, Li F (2006). The c-Myc target gene network. Semin Cancer Biol.

[21] Eilers M, Eisenman RN (2008). Myc’s broad reach. Genes Dev.

[22] Stine ZE, Walton ZE, Altman BJ, Hsieh AL, Dang CV (2015). MYC, metabolism, and cancer. Cancer Discov.

[23] Dang CV (2012). MYC on the path to cancer. Cell.

[24] El Baroudi M, Cora D, Bosia C, Osella M, Caselle M (2011). A curated database of miRNA mediated feed-forward loops involving MYC as master regulator. PloS ONE.

[25] Musilova K, Mraz M (2015). MicroRNAs in B-cell lymphomas: how a complex biology gets more complex. Leukemia.

[26] Pan L, Gong Z, Zhong Z, Dong Z, Liu Q, Le Y (2011). Lin-28 reactivation is required for let-7 repression and proliferation in human small cell lung cancer cells. Mol Cell Biochem.

[27] Pekarsky Y, Balatti V, Croce CM (2018). BCL2 and miR-15/16: from gene discovery to treatment. Cell Death Differ.

[28] Horie M, Kaczkowski B, Ohshima M, Matsuzaki H, Noguchi S, Mikami Y (2017). Integrative CAGE and DNA methylation profiling identify epigenetically regulated genes in NSCLC. Mol Cancer Res.

[29] Li S, Zhu J, Fu H, Wan J, Hu Z, Liu S (2012). Hepato-specific microRNA-122 facilitates accumulation of newly synthesized miRNA through regulating PRKRA. Nucleic Acids Res.

[30] Li S, Fu H, Wang Y, Tie Y, Xing R, Zhu J (2009). MicroRNA-101 regulates expression of the v-fos FBJ murine osteosarcoma viral oncogene homolog (FOS) oncogene in human hepatocellular carcinoma. Hepatology.

[31] Zhu Y, Gu J, Li Y, Peng C, Shi M, Wang X (2018). MiR-17-5p enhances pancreatic cancer proliferation by altering cell cycle profiles via disruption of RBL2/E2F4-repressing complexes. Cancer Lett.

[32] Zhu J, Liu S, Ye F, Shen Y, Tie Y, Zhu J (2015). Long noncoding RNA MEG3 interacts with p53 protein and regulates partial p53 target genes in hepatoma cells. PloS ONE.

[33] Zhang L, Wei N, Cui Y, Hong Z, Liu X, Wang Q (2018). The deubiquitinase CYLD is a specific checkpoint of the STING antiviral signaling pathway. PLoS Pathog.

[34] Tang Z, Li C, Kang B, Gao G, Li C, Zhang Z (2017). GEPIA: a web server for cancer and normal gene expression profiling and interactive analyses. Nucleic Acids Res.

[35] Hu H, Liu JM, Hu Z, Jiang X, Yang X, Li J (2018). Recently Evolved Tumor Suppressor Transcript TP73-AS1 Functions as Sponge of Human-Specific miR-941. Mol Biol Evolution.

[36] Bolger AM, Lohse M, Usadel B (2014). Trimmomatic: a flexible trimmer for Illumina sequence data. Bioinformatics.

[37] Kim D, Langmead B, Salzberg SL (2015). HISAT: a fast spliced aligner with low memory requirements. Nat Methods.

[38] Aken BL, Ayling S, Barrell D, Clarke L, Curwen V, Fairley S (2016). The Ensembl gene annotation system. Database: J Biol Databases Curation.

[39] Robinson MD, McCarthy DJ, Smyth GK (2010). edgeR: a Bioconductor package for differential expression analysis of digital gene expression data. Bioinformatics.

[40] Hu HY, Guo S, Xi J, Yan Z, Fu N, Zhang X (2011). MicroRNA expression and regulation in human, chimpanzee, and macaque brains. PLoS Genet.

[41] Hu HY, Yan Z, Xu Y, Hu H, Menzel C, Zhou YH (2009). Sequence features associated with microRNA strand selection in humans and flies. BMC Genomics.

[42] Langmead B, Trapnell C, Pop M, Salzberg SL (2009). Ultrafast and memory-efficient alignment of short DNA sequences to the human genome. Genome Biol.

[43] Hu HY, He L, Fominykh K, Yan Z, Guo S, Zhang X (2012). Evolution of the human-specific microRNA miR-941. Nat Commun.

[44] Weinstein JN, Collisson EA, Mills GB, Shaw KR, Ozenberger BA, Cancer Genome Atlas Research N (2013). The Cancer Genome Atlas Pan-Cancer analysis project. Nat Genet.

[45] Consortium GT (2013). The Genotype-Tissue Expression (GTEx) project. Nat Genet.

[46] Consortium GT (2015). Human genomics. The Genotype-Tissue Expression (GTEx) pilot analysis: multitissue gene regulation in humans. Science.

[47] Li Y, Casey SC, Choi PS, Felsher DW (2014). miR-17-92 explains MYC oncogene addiction. Mol Cell Oncol.

[48] Wang Z, Liu M, Zhu H, Zhang W, He S, Hu C (2010). Suppression of p21 by c-Myc through members of miR-17 family at the post-transcriptional level. Int J Oncol.

[49] Tang Q, Zou Z, Zou C, Zhang Q, Huang R, Guan X (2015). MicroRNA-93 suppress colorectal cancer development via Wnt/beta-catenin pathway downregulating. Tumour Biol: J Int Soc Oncodev Biol Med.

[50] Abreu FB, Liu X, Tsongalis GJ (2017). miRNA analysis in pancreatic cancer: the Dartmouth experience. Clin Chem Lab Med.

[51] Ali S, Almhanna K, Chen W, Philip PA, Sarkar FH (2010). Differentially expressed miRNAs in the plasma may provide a molecular signature for aggressive pancreatic cancer. Am J Transl Res.

[52] Dellago H, Bobbili MR, Grillari J (2017). MicroRNA-17-5p: at the crossroads of cancer and aging - a mini-review. Gerontology.

[53] Chen S, Chen X, Sun KX, Xiu YL, Liu BL, Feng MX (2016). MicroRNA-93 promotes epithelial-mesenchymal transition of endometrial carcinoma cells. PloS ONE.

[54] Luan Y, Chen M, Zhou L (2017). MiR-17 targets PTEN and facilitates glial scar formation after spinal cord injuries via the PI3K/Akt/mTOR pathway. Brain Res Bull.

[55] Li C, Lyu J, Meng QH (2017). MiR-93 promotes tumorigenesis and metastasis of non-small cell lung cancer cells by activating the PI3K/Akt pathway via inhibition of LKB1/PTEN/CDKN1A. J Cancer.

[56] Ohta K, Hoshino H, Wang J, Ono S, Iida Y, Hata K (2015). MicroRNA-93 activates c-Met/PI3K/Akt pathway activity in hepatocellular carcinoma by directly inhibiting PTEN and CDKN1A. Oncotarget.

[57] Carnero A, Blanco-Aparicio C, Renner O, Link W, Leal JF (2008). The PTEN/PI3K/AKT signalling pathway in cancer, therapeutic implications. Curr Cancer Drug Targets.

[58] Anfossi S, Fu X, Nagvekar R, Calin GA (2018). MicroRNAs, regulatory messengers inside and outside cancer cells. Adv Exp Med Biol.

[59] Nesbit CE, Tersak JM, Prochownik EV (1999). MYC oncogenes and human neoplastic disease. Oncogene.

[60] Mihailovich M, Bremang M, Spadotto V, Musiani D, Vitale E, Varano G (2015). miR-17-92 fine-tunes MYC expression and function to ensure optimal B cell lymphoma growth. Nat Commun.

[61] Luscher B, Vervoorts J (2012). Regulation of gene transcription by the oncoprotein MYC. Gene.

