# Supplementary material for: The MYEOV-MYC association promotes oncogenic miR-17/93-5p expression in pancreatic ductal adenocarcinoma
Source: Cell Death Dis. 2021 Dec 20;13(1):15. doi: 10.1038/s41419-021-04387-z (PMC8688437; doi:10.1038/s41419-021-04387-z)
Supplement: Supplementary file 1 — Fig. S1-3 [file 41419_2021_4387_MOESM1_ESM.docx]

**Supplementary Figures for**

**The MYEOV-MYC association promotes oncogenic miR-17/93-5p expression in pancreatic ductal adenocarcinoma**

Hongzhang Shen 1^#^, Fuqiang Ye 3^#^, Dongchao Xu 1, Liangliang Fang 4, Xiaofeng Zhang 1*, Juanjuan Zhu 2*

^1^Department of Gastroenterology, Affiliated Hangzhou First People’s Hospital, Zhejiang University School of Medicine, Hangzhou, 310006, China

^2^School of Life Science and Technology, China Pharmaceutical University, Nanjing, 211198, China

^3^ Huadong Research Institute for Medicine and Biotechniques, Nanjing, 210002, China

^4^The First School of Clinical Medicine, Nanjing Medical University, 211166, China

^#^ These authors contributed equally to this work

*Xiaofeng Zhang: 837837@zju.edu.cn Juanjuan Zhu: zhujuanjuan1204@126.com


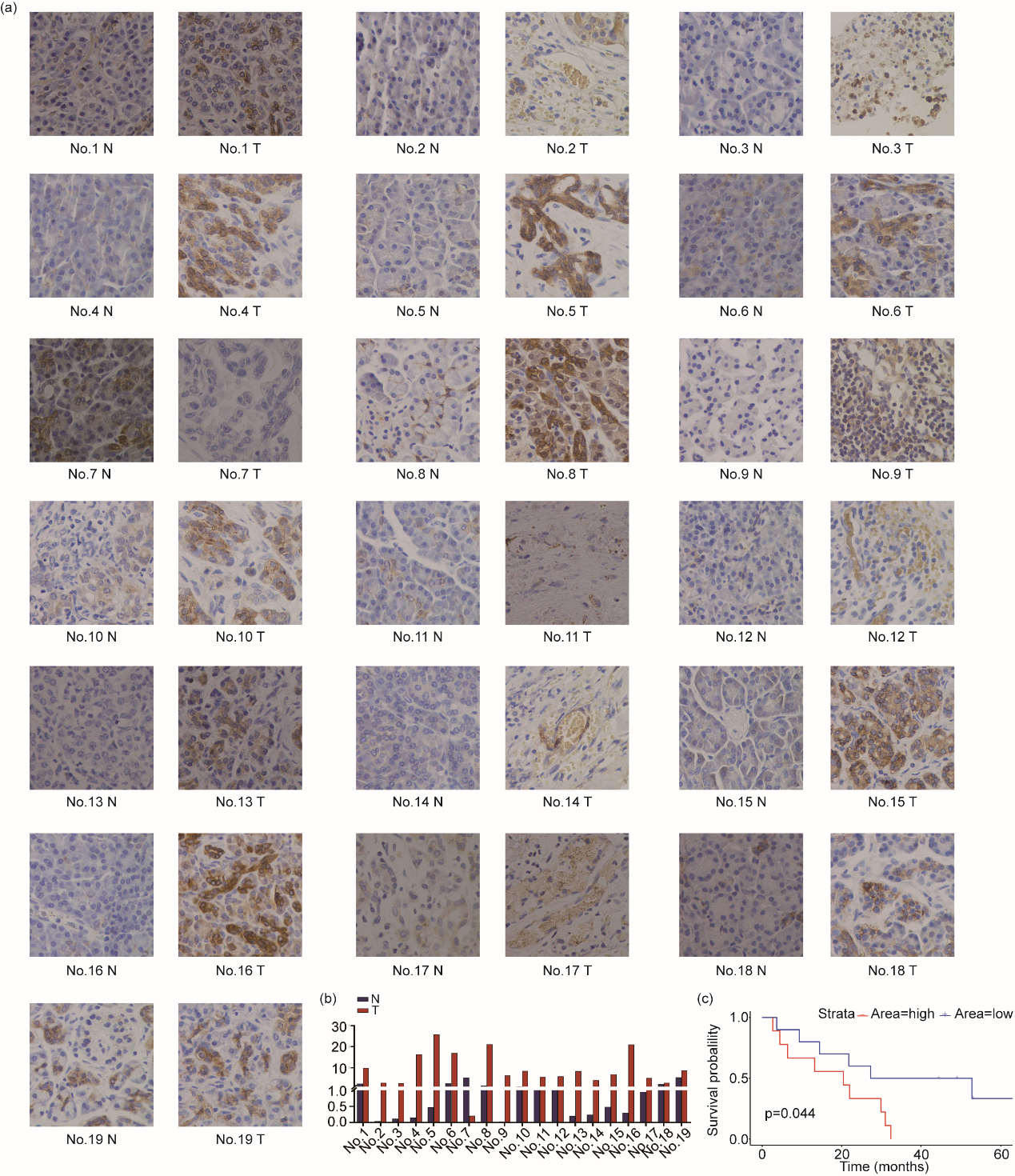


**Fig. S1** (a) IHC staining analyses of 19 FFPE PDAC and NCP tissues using an anti-MYEOV antibody (400× magnification). (b) Semi-quantitative analysis of fig S1a. (c) Kaplan-Meier curves of overall survival of patients with PDAC based on MYEOV expression in the immunohistochemical staining results using the log-rank test. The dotted lines indicate the 95% confidence intervals for survival percentage at each time point as obtained by survival analysis. N, non-cancerous pancreas; T, pancreatic tumor.


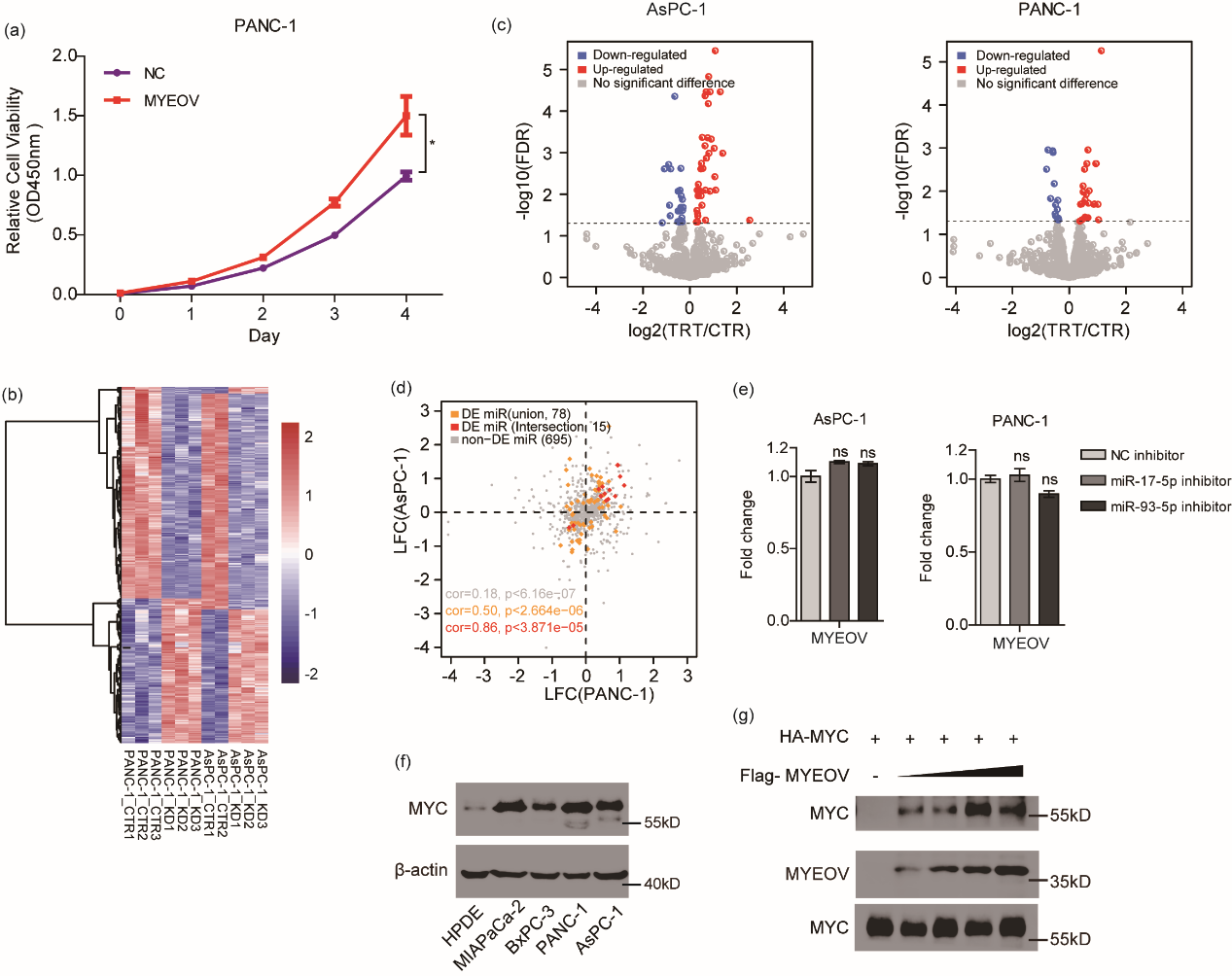


**Fig. S2** (a) Elevated cell viability of PANC-1 cells after MYEOV overexpression. (b) Heatmap of differentially expressed genes due to MYEOV knockdown in two PC cell lines (AsPC-1 and PANC-1). (c) The miRNA expression changes in two PC cell lines (AsPC-1 and PANC-1) after MYEOV knockdown. Significantly induced (red) and repressed miRNAs (blue) after MYEOV knockdown in AsPC-1 and PANC-1 cells (negative binominal test, FDR<0.05). The x-axis represents the LFC between MYEOV-knockdown and wild-type PC cells. The y-axis represents the magnitude of significance as measured by the FDR of the negative binominal test in -log10 scale. (d) The correlation of miRNA expression changes after MYEOV knockdown between AsPC-1 and PANC-1 cells. Seventy-eight points in orange showed the union set of differentially expressed miRNAs of the two PC cell lines. Fifteen points in red show the intersection set of differentially expressed miRNAs of the two PC cell lines. The remaining 695 expressed miRNAs are shown in grey. Both the a-axis and y-axis represent miRNA expression fold changes in log2 scale between MYEOV-knockdown and cognate wild-type PC cells. (e) The qPCR-based expression levels of MYEOV upon miR-17-5p or miR-93-5p inhibition. (f) Comparison of MYC expression levels in the PC cell lines with those observed in normal pancreatic epithelial cells by Western blot. (g) The association of MYEOV with MYC is dose-dependent.


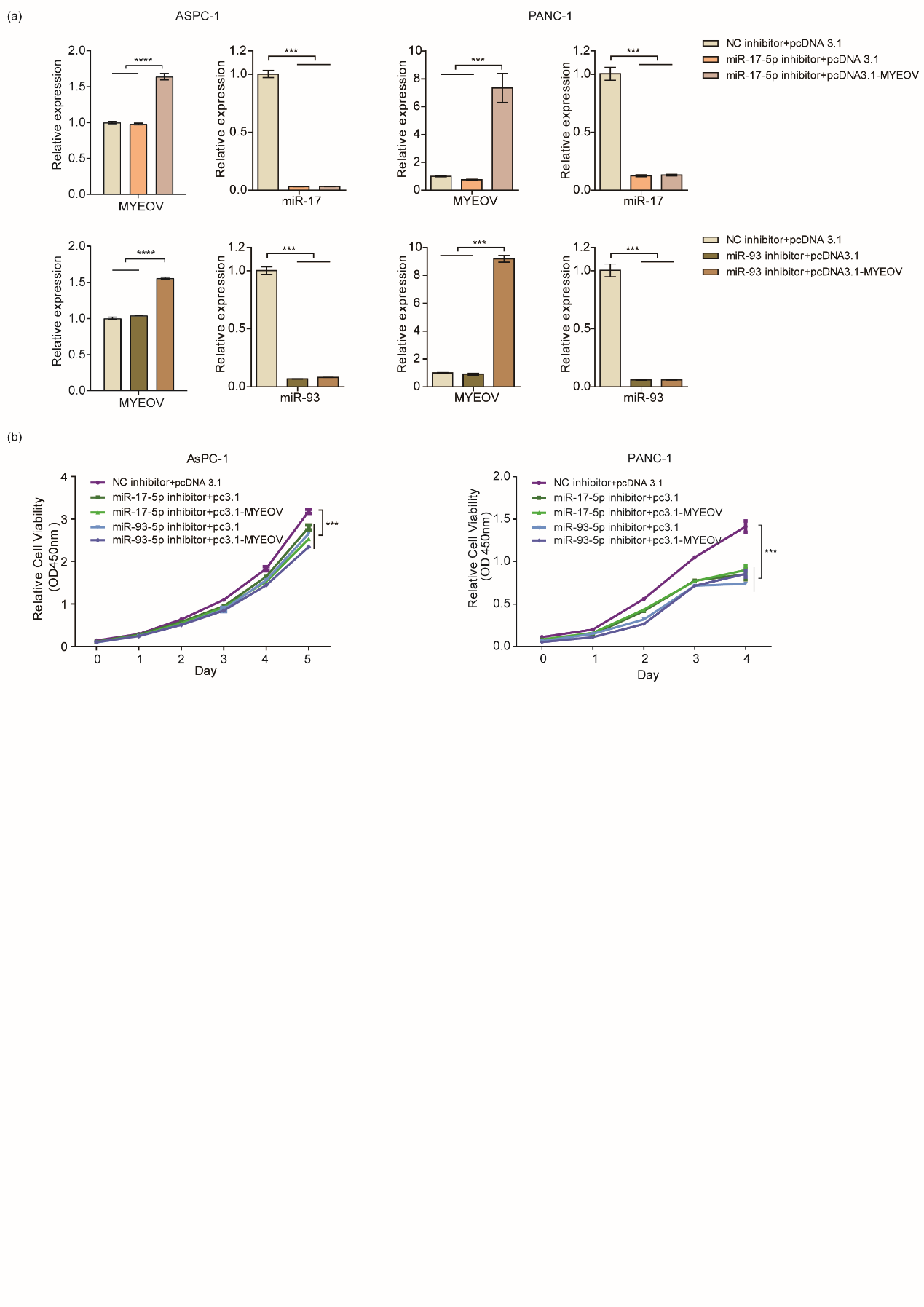


**Fig. S3** Alteration of cell proliferation in miR-17/93-5p-inhibitory PDAC after MYEOV overexpression. (a) qPCR-based expression levels of MYEOV, miR-17-5p and miR-93-5p. (b) Cell proliferation as measured by CCK-8 assays. All *n* ≥ 3; bar, SEM; *P < 0.05, **P < 0.01 and ***P < 0.001 compared with NC; Student’s t-test.
